# The relation of 25-hydroxy vitamin D concentrations to liver histopathology, seasonality and baseline characteristics in chronic hepatitis C virus genotype 2 or 3 infection

**DOI:** 10.1371/journal.pone.0237840

**Published:** 2020-08-21

**Authors:** Jesper Waldenström, Kristina Nyström, Staffan Nilsson, Gunnar Norkrans, Magdalena Ydreborg, Nina Langeland, Kristine Mørch, Johan Westin, Martin Lagging

**Affiliations:** 1 Department of Infectious Diseases, Institute of Biomedicine, Sahlgrenska Academy, University of Gothenburg, Gothenburg, Sweden; 2 Region Västra Götaland, Sahlgrenska University Hospital, Department of Clinical Microbiology, Gothenburg, Sweden; 3 Department of Mathematical Sciences, Chalmers University of Technology, Gothenburg, Sweden; 4 Department of Medicine, Haukeland University Hospital, Bergen, Norway; 5 Department of Clinical Science, University of Bergen, Bergen, Norway; FIOCRUZ, BRAZIL

## Abstract

**Background and objectives:**

The hydroxylation to 25-hydroxy vitamin D (25(OH)D) occurs in the liver and the impact of liver disease on vitamin D is unclear. This study evaluated the relationship between vitamin D concentrations and hepatic histopathology, seasonality and patient characteristics in well-characterized patients having undergone a liver biopsy.

**Method:**

25(OH)D was measured *post-hoc* in pre-treatment serum from 331 North European patients with chronic HCV genotype 2 or 3 infection (NORDynamIC study). Liver biopsies were scored for fibrosis and inflammation according to the Ishak protocol, and graded for steatosis. Non-invasive markers of hepatic fibrosis as well as baseline viral and host characteristics, including genetic polymorphisms rs2228570, rs7975232, and rs10877012 were also evaluated.

**Results:**

Mean 25(OH)D concentration was 59 ±23 nmol/L, with 41% having values <50 nmol/L and 6% were <30 nmol/L. 25(OH)D correlated with fibrosis (r = -0.10, p ≤0.05) in univariate but not in multivariate analyses. No association was observed between 25(OH)D and hepatic inflammation, but with steatosis in HCV genotype 2 infected patients. None of the genetic polymorphisms impacted on 25(OH)D levels or fibrosis. 25(OH)D levels were significantly inversely correlated to BMI (r = -0.19, p = 0.001), and was also associated with season and non-Caucasian ethnicity.

**Conclusion:**

Fibrosis was not independently associated with 25(OH)D concentration and no association was seen with hepatic inflammation, but HCV genotype 2 infected patients with moderate-to-severe steatosis had lower 25(OH)D levels compared to those without steatosis. A high percentage had potential risk of 25(OH)D deficiency, and BMI, seasonality and ethnicity were independently associated with 25(OH)D as previously reported.

## Introduction

Vitamin D is essential for bone mineralization by regulating calcium and phosphate levels in blood, and deficiency can lead to rickets and osteomalacia [1]. Lately increasing evidence also suggests importance of vitamin D regarding infectious diseases, malignancies, autoimmune diseases, obesity, type 2 diabetes, liver diseases and risk of death [2, 3], although these latter associations have been questioned, especially regarding possible causality [4].

The majority of vitamin D is endogenously synthesized from D7-dehydrocholesterol upon exposure of skin to ultraviolet (UV) radiation, but can also be ingested orally by vitamin D rich nutrients. As UV radiation in the northern latitudes is insufficient for production during winter, stored vitamin D, sufficient dietary intake, and/or supplementation are needed for maintenance of adequate levels during this season [5]. Vitamin D requires two successive hydroxylation reactions to be activated: i) in the liver by the enzyme cholecalciferol 25-hydroxylase to 25-hydroxy vitamin D (25(OH)D) also known as calcifediol and ii) mainly in the kidneys by cytochrome p450 27B1 (CYP27B1; also known as 1-alpha-hydroxylase) to 1,25-dihydroxy vitamin D also referred to as calcitriol. Calcitriol signals through the vitamin D receptor (VDR), influences the expression of a multitude of genes, and is expressed in most human cell types indicating a broader effect than regulation of bone mineralization [6]. Calcitriol has a short half-life and varies in concentration. In contrast 25(OH)D is stable and more accurately reflects vitamin D status, and therefore is preferred for detecting vitamin D deficiency [7]. Vitamin D is fat soluble, and higher body mass index (BMI) is known to associate with lower systemic 25(OH)D concentrations [2].

The definition of vitamin D deficiency is controversial. The Institute of Medicine (IOM) has defined two cut-offs: i) ≥50 nmol/L 25(OH)D where deficiency is highly unlikely, and ii) <30 nmol/L where it is likely [8]. The Endocrine Society has an additional cut-off at <75 nmol/L, with concentrations between 50–75 considered insufficient [9]. In this study we used the definition by IOM [10].

Vitamin D reportedly affects both innate and adaptive immunity, and has been extensively studied in diseases entailing inflammation [11], including chronic hepatitis C virus (HCV) infection. Vitamin D has been reported to have an *in vitro* antiviral effect on HCV [12, 13] and previous publications reports lower likelihood of achieving a cure, i.e. sustained virologic response (SVR), following interferon-based therapy in the presence of low systemic concentrations of 25(OH)D [14–16]. A recent meta-analysis demonstrates a significant association between 25(OH)D concentration and fibrosis. The analysis was composed of twelve studies of which eight could observe an association, whereas four could not. Importantly only one study reported having HCV genotype 2 or 3 infected participants [17]. Many studies in other chronic liver diseases have also noted decreasing vitamin D levels with cirrhosis [18]. Low vitamin D levels has also been linked with steatosis and liver inflammation [10, 19].

Several genetic polymorphisms associated with 25 (OH) vitamin D signaling and metabolism have been studied in patients with chronic hepatitis C. The Vitamin D receptor (VDR) contains several and among them a combined genotype, in strong linkage disequilibrium, referred to as CCA haplotype. This haplotype comprises rs1544410 (BsmI) C, rs7975232 (ApaI) C and rs731236 (TaqI) A alleles and has been associated with liver fibrosis progression and response to interferon and ribavirin treatment [20, 21]. Another VDR single-nucleotide polymorphism (SNP) in exon 2, rs2228570 (FokI, aka rs10735810), is located within a start codon where the presence of a C allele instead of T allele leads to the initiation of translation at an alternative site, resulting in a shorter, reportedly less active receptor [22]. There are also several single nucleotide polymorphisms (SNPs) associated with the metabolism of vitamin D, of which rs10877012 is situated in the CYP27B-1260 gene promotor. In a study by Lange et al, the A allele was associated with higher serum concentration of the active form of vitamin D, 1.25(OH)D and SVR [23].

As the first hydroxylation step activating vitamin D to 25(OH)D occurs in the liver and as the impact of hepatic histopathology on 25(OH)D levels remains unclear, this study thus aimed at evaluating the vitamin D concentrations in relation to liver histopathology, as well as other baseline characteristics, in very well-characterized patients enrolled in a north European multicenter HCV therapeutic trial (NORDynamIC), where pre-treatment liver biopsies were mandatory [24] and where a relatively large proportion had advanced liver fibrosis.

## Method

### Study design and population

The study population was derived from the NORDynamIC study in which 382 treatment-naïve, HCV genotype 2 or 3 chronically infected patient were enrolled. The study was conducted between February 2004 and November 2005 at 31 centers in Sweden, Denmark, Finland and Norway, located between latitudes 55–62° [24]. All patients had compensated liver disease, were adults and had no co-infection with hepatitis B virus or human immunodeficiency virus (HIV). In this phase III, open label, randomized, multicenter, investigator-initiated treatment study patients were randomized to receive either 12 or 24 weeks of treatment with 180 μg of peg-interferon (Peg-IFN) α-2a once weekly and 800 mg/day ribavirin. Pre-treatment serum samples were stored at -80°C. Pre-treatment liver biopsies were mandatory and were available for assessment for 354 of 382 patients. Pre-treatment serum samples for 25(OH)D analysis were available from 331 of these 354 patients, which comprised the study population. Baseline characteristics and treatment response are detailed in Table 1. Forty-three patients (13% of the total study population) had cirrhosis, 313 of 331 patients were of Caucasian ethnicity, 60% of participants were male. All concomitant medication was documented through the study, and vitamin D supplementation was only used by four patients and only in multivitamin tablets. Child-Pugh B and C cirrhotic patients, i.e. decompensated cirrhosis, were excluded from the NORDynamIC study as interferon therapy is contraindicated in these patients.

**Table 1:** Characteristics of the 331 patients included in the study and differences in characteristics according to 25 (OH) vitamin D sufficiency, potential risk of deficiency and risk of deficiency.

Feature	All patients	>50 nmol/L, sufficiency	30–50 nmol/L, potentially at risk of deficiency	<30 nmol/L, risk of deficiency	
	n = 331	n = 197 (59.5%)	n = 113 (34.1%)	n = 21 (6.3%)	Unadjusted *P-value*
Epidemiological features					
Age, mean (SD), years	41.9 (10.8)	41.7 (11.0)	41.4 (10.9)	46.3 (8.6)	0.16
Sex male n(%)	200 (60.4)	116 (59)	71 (63)	13 (62)	0.78
BMI, mean (SD), kg/m2	25.8 (4.4)	25.4 (3.8)	26.3 (4.8)	27.6 (6.2)	**0.04**
Ethnicity, non-Caucasian n(%)	16 (5)	6 (3)	6 (5)	4 (19)	**0.003**
Characteristics of HCV infection					
Estimated duration of infection, median (range), years	13 (1–60)	13.5 (1–48)	13.5 (1–41)	10 (1–60)	0.95
HCV-RNA at baseline, mean (SD), log10 IU/mL	6.0 (0.9)	6.1 (0.8)	6.0 (0.9)	5.8 (1.0)	0.33
HCV genotype n (%), genotype 2/3/2+3	94/236/1 (28.4/71.3/0.3)	51/145/1 (26/75/0)	38/75 (34/66)	5/16 (24/76)	0.32
Liver fibrosis Ishak stage, 0/1/2/3/4/5/6, n	12/46/100/86/44/19/24	7/25/66/54/22/11/12	5/20/29/27/17/6/9	0/1/5/5/5/2/3	0.06
Cirrhosis, n(%)	43 (13)	23 (12)	15 (13)	5 (24)	0.29
Liver interface hepatitis Ishak grade, 0/1/2/3/4, n	20/104/127/74/6	15/58/79/43/2	4/41/39/27/2	1/5/9/4/2	0.61
Liver lobular inflammation Ishak grade, 0/1/2/3/4, n	1/72/194/61/3	1/41/121/32/2	0/25/66/21/1	0/6/7/8/0	0.69
Liver portal inflammation Ishak grade, 0/1/2/3/4, n	14/154/135/28/0	9/96/71/21/0	5/48/55/5/0	0/10/9/2/0	0.82
Liver steatosis score, 0/1/2/3, n	113/128/54/36	77/68/33/19	31/54/15/15	5/6/6/4	0.07
APRI-score, mean (SD), score	1.1 (1.2)	1.0 (1.1)	1.0 (1.0)	1.52 (2.1)	0.18
Treatment regimens and response					
12/24week Peg-IFN + ribavirin, n(%)	163/168 (49.2/50.8)	103/94 (52/48)	50/63 (44/56)	10/11 (48/52)	0.39
SVR rate, n(%)	224 (68)	132 (67)	82 (73)	10 (48)	0.08
Season					
Sunny season at sampling n(%)	226 (68)	143 (73)	71 (63)	12 (57)	0.10
Host genetics^a^					
Vitamin D3 receptor (VDR) Gene rs2228570 (Fok1), CC/CT/TT, n(%)	118/147/50 (37/47/16)	73/88/31 (38/46/16)	38/53/15 (36/50/15)	7/6/4(35/35/24)	0.78
Vitamin D3 receptor (VDR) Gene rs7975232 (ApaI), CC/AC/AA, n(%)	53/152/108 (17/49/34)	35/85/71 (18/45/37)	17/56/32 (16/53/30)	1/11/5 (6/65/29)	0.34
CYP27B-1260 Gene Promotor rs10877012, TT/TG/GG, n(%)	127/149/39 (40/47/12)	80/90/22 (42/47/11)	39/53/14 (37/50/13)	8/6/3 (47/35/18)	0.76
Serum 25 (OH)D levels					
Serum 25 (OH)D, mean (SD), nmol/L	58.9 (22.5)				
<25 nmol/L, n(%)	9 (2.7)				
> 75 nmol/L n(%)	75 (22.7)				

HCV, hepatitis C virus; APRI, AST to platelet ratio; Peg-IFN, pegylated interferon; SD, standard deviation; SVR, sustained virologic response.^a b^Only patients with Caucasian ethnicity were included in the analysis.

### Assessments of liver biopsies

The liver biopsies were centrally staged for fibrosis in a blinded fashion according to the Ishak protocol by two independent experienced observers (J.We. and M.L.). When diverging results were obtained, renewed assessments were performed and a consensus score was agreed upon. Ishak has seven different stages of fibrosis, i.e. stages 0–6. In this study, absence of fibrosis is defined as Ishak stage 0, mild fibrosis as stage 1–2, moderate fibrosis as stage 3–4 and cirrhosis as stage 5–6. Inflammation was assessed using the Ishak protocol and are presented as grade of interface hepatitis (0–4), lobular inflammation (0–4), portal inflammation (0–4) or a sum of all these inflammation scores. Steatosis was also graded as absent (grade 0), mild (less than 30% of hepatocytes involved, grade 1), moderate (30–70% of hepatocytes involved, grade 2) or severe (>70% of hepatocytes involved, grade 3).

### HCV RNA quantification

HCV RNA was determined by qRT-PCR of plasma using Cobas AmpliPrep/COBAS TaqMan HCV Test (Roche Diagnostics, Branchburg, NJ). Sustained virologic response was defined as negative HCV-RNA 24 weeks after completion of therapy.

### Measurement of 25(OH)D

Fasting serum samples was drawn from patients before treatment initiation. For determination of serum concentration of 25(OH)D, a commercially available chemiluminiscence assay, Architect 25-hydroxyvitamin D assay (Abbot diagnostics, Illinois USA) was used. The assay does not discriminate between the two forms of vitamin D, i.e. Vitamin D3 cholecalciferol and vitamin D2 ergocalciferol, and therefore measures the total amount of 25(OH)D.

### Vitamin D related single nucleotide polymorphism assays

Polymorphisms were determined in serum by allelic discrimination real-time PCR using predesigned Taq-Man SNP Assays (Life Technologies, California USA) for rs7975232, rs2228570 and rs10877012 according to manufacturer’s instructions. rs7975232 (C>A) is also known as ApaI and located in the VDR gene. rs2228570 (C>T) is known as FokI, is also located in the VDR gene. rs10877012 (G>T) is located in the promotor region of the gene for CYP27B1.

### Statistical methods

Continuous variables are presented as either mean with standard deviation or median with range based on normal distribution and categorical values as frequencies with percentage. Liver biopsy scores are presented as number of patients with a certain stage, grade, or score. Differences in categorical variables were compared using either the *χ*2, Fisher’s exact test or binominal regression. The ordinal data has been used as metric data, after consideration in multiple regression, and also in some correlation analysis. Correlations were done with Pearson’s correlation for continuous and ordinal variables with normal distribution. Differences between groups with continues and ordinal data were done with one-way analysis of variance (ANOVA) test together with Tukey’s post hoc test. When comparing two groups a student T-test was done. In the multivariate analysis for the association between Cirrhosis and vitamin D, a multiple binominal logistic regression was performed. Multiple linear regression was done to to evaluate variables in relation to fibrosis, steatosis and 25(OH)D. Parameters with a p value of <0.1 were included in the multiple regression analysis. Single nucleotide polymorphisms were coded as 1, 2 or 3 in these analyses. For analysis of genetic data only Caucasian patients were included in the analysis. All statistical analyses were performed using the IBM SPSS statistics version 19 (IBM Corporation, Somers, NY) software package. All P-values are two sided and values of <0.05 were considered statistically significant.

### Season and UVB radiation data

Data of UVB radiation from the Swedish Metrological and Hydrological Institute (SMHI; Norrköping, Sweden) was used to choose two periods in which the sun exposure differed the most [25]. For Sweden, the least radiation was seen in the period from November to the end of February. Since Norway, Finland and Denmark to a great extent overlap in latitude with Sweden, this data was used for all patients.

### Ethical considerations

The Regional Ethics Review Board in Gothenburg approved the study. All patients signed informed consent. The study is registered at the NIH trial registry (ClinicalTrials.gov Identifier: NCT00143000).

## Results

### Serum 25(OH)D levels

Mean serum 25(OH)D concentration was 59 nmol/L, and there was a high percentage, 41%, of patients with levels defined as potentially having a risk of deficiency, i.e. <50 nmol/L (equivalent to 20 ng/L), but only 6% had a high risk of deficiency defined as <30 nmol/L (equivalent of 12 ng/L). Interestingly, only 23% of patients had levels >75nmol/L, which the endocrinology society defines as sufficient levels, Table 1. Lower 25(OH)D level groups were significantly associated with non-Caucasian ethnicity, higher fibrosis stage, and higher BMI in univariate analyses, Table 1. 25(OH) D concentration was not significantly different based on genetic variations in rs2228570, rs7975232 and rs10877012, Table 2. Since ethnicity might have impacted the result a separate analysis regarding baseline characteristics and fibrosis as well as 25 (OH) D concentrations was performed with the only major difference being an association between viral genotype and 25 (OH) D concentration, S1 Table.

**Table 2:** Associations of baseline characteristics with 25 (OH) D concentrations and fibrosis.

Feature	Associations of Baseline Characteristics and 25 (OH) D concentrations						Associations of Baseline Characteristics and fibrosis					
	B-value	β-value	*P-value*	Adjusted B-value	Adjusted β-value	Adjusted *P-value*	B-value	β-value	*P-value*	*Adjusted B-value*	Adjusted β-value	Adjusted *P-value*
Epidemiological features												
Age, years	-0.12	-0.06	0.3				0.05	0.44	**<0.001**	0.05	0.34	**<0.001**
Sex (female)	0.03	0.00	1.0				-0.40	-0.13	**0.02**	-0.13	-0.04	0.4
BMI, kg/m2	-0.98	-0.19	**0.001**	-0.97	-0.19	**0.001**	0.06	0.19	**0.001**	0.05	0.14	**0.005**
Ethnicity, (non-Caucasian)	-14.42	-0.14	**0.01**	-15.86	-0.15	**0.005**	-0.16	-0.02	0.7			
Characteristics of HCV infection												
HCV-RNA at baseline, log10 IU/mL	1.73	0.07	0.23				0.34	0.20	**<0.001**	0.06	0.04	0.5
HCV genotype, genotype 2/3	3.88	0.08	0.16				-0.1 1	-0.03	0.5			
Liver firosis Ishak, score	-1.69	-0.11	**≤0.05**	-1.47	-0.10	0.08						
Liver Ishak inflammation, sum of score	-0.92	-0.08	0.16				0.54	0.70	**<0.001**^**a**^			
Liver Ishak steatosis, score	-2.05	-0.09	0.11				0.34	0.23	**<0.001**	0.03	0.02	0.7
APRI-score (log10)	-1.89	-0.03	0.6				2.05	0.49	**<0.001**	1.69	0.41	**<0.001**
Season												
Sunny season at sampling	8.03	0.17	**0.002**	8.23	0.17	**0.001**						
Host genetics ^**b**^												
Vitamin D3 receptor Gene rs2228570 CC/CT/TT	-0.38	-0.01	0.83				-0.14	-0.06	0.3			
Vitamin D3 receptor Gene rs7975232 CC/AC/AA	-1.63	-0.05	0.37				-0.16	-0.07	0.2			
CYP27B-1260 Gene Promotor rs10877012 TT/TG/GG	-2.07	-0.06	0.27				-0.01	-0.006	0.9			
Serum 25 (OH)D levels												
Serum 25 (OH)D, nmol/L							**-0.01**	**-0.11**	**≤0.05**	-0.00	-0.02	0.6

HCV, hepatitis C virus; APRI, sat to platelet ratio. ^a^Excluded from multiple regression due to multicollinearity. ^b^Only patients with Caucasian ethnicity were included in the analysis.

### Liver fibrosis

**Fig 1:**
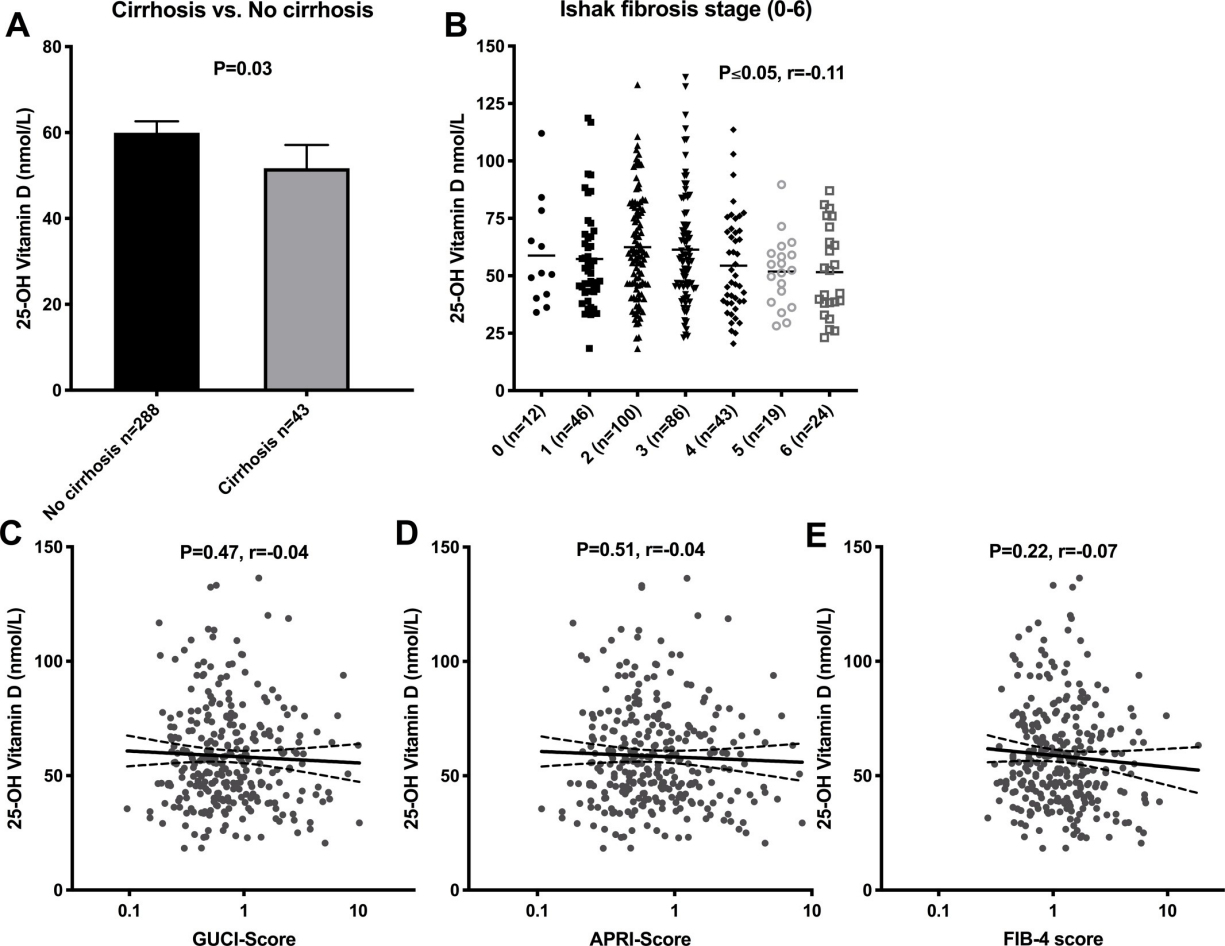
25-hydroxy vitamin D and liver fibrosis. Bar chart for 25-hydroxy vitamin D and liver cirrhosis (A) and scatter plots for 25-hydroxy vitamin D and Ishak fibrosis stage (B) fibrosis indices GUCI-score (C), APRI-score (D) and Fib-4 score (E). Mean concentration and 95% confidence interval is shown. Statistical significance determined by Student T test and Pearson’s test.

A significant, albeit weak correlation was observed for Ishak fibrosis stage and 25(OH)D concentrations (r = -0.10, p ≤0.05). Fibrosis stage and cirrhosis did not significantly differ between 25(OH)D groups, Table 1. However, mean 25(OH)D level was significantly lower in cirrhotic compared to non-cirrhotic patients, although this difference was modest (52 ±18 vs. 60 ±23 nmol/L, p = 0.03), Fig 1A. The association was no longer significant in a binominal logistic regression analysis, including season at sampling, BMI, gender, APRI-score and age, indicating that 25(OH)D level was not independently associated with cirrhosis in this study, Table 3. Similarly, 25(OH)D did not correlate with the non-invasive fibrosis indices GUCI, APRI, or FIB-4, Fig 1C–1E. The fibrosis score was not significantly different based on genetic variations in rs2228570, rs7975232 and rs10877012, Table 2.

**Table 3:** Differences in baseline characteristics according to cirrhosis.

Feature	Cirrhosis	No cirrhosis	Odds ratio (95% CI)	Unadjusted *P-value*	Adjusted odds ratio (95% CI)	Adjusted *P-value*
	n = 43	n = 288				
Epidemiological features						
Age, mean (SD), years	50.7 (10.5)	40.6 (8.9)	1.1 (1.1–1.1)	**<0.0001**	1.1 (1.1–1.2)	**<0.0001**
Sex male n(%)	32 (74)	168 (58)	2.1 (1.0–4.3)	**0.05**	2.2 (0.9–5.6)	0.09
BMI, mean (SD), kg/m2	28.2 (3.9)	25.5 (4.3)	1.1 (1.1–1.2)	**0.0002**	1.1 (1.0–1.3)	**0.004**
Characteristics of HCV infection						
HCV-RNA at baseline, mean SD, log10 IU/mL	6.3 (0.7)	6.0 (0.9)	1.4 (0.9–2.1)	0.15		
HCV genotype n (%), genotype 2/3	13/30 (30/70)	81/206 (28/72)	1,1 (0.5–2.2)	0.79		
APRI-score, mean (SD), score	2.1 (1.6)	0.9 (1.0)	1.8 (1.4–2.2)	**<0.0001**	1.7 (1.3–2.1)	**<0.0001**
Serum 25 (OH)D levels						
Serum 25 (OH)D, mean (SD), nmol/L	51.7 (17.6)	60.0 (23.0)	0.7 (0.5–1.0)*	**0.03**	0.8 (0.5–1.3)*	0.35

HCV, hepatitis C virus; APRI, ast to platelet ratio; SD, standard deviation. *Odds ratio calculated from standard score of 25 (OH)D.

### Liver inflammation

**Fig 2:**
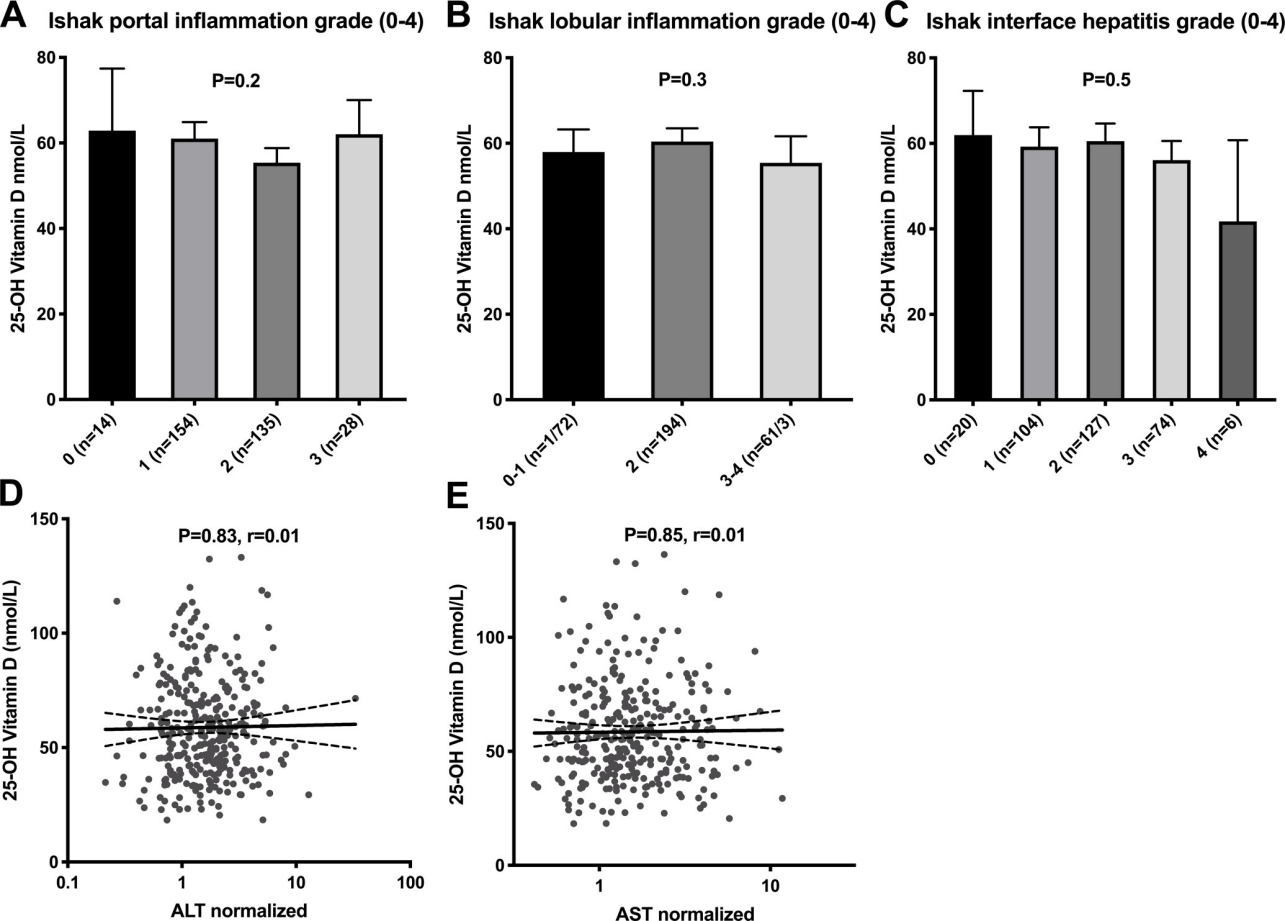
25-hydroxy vitamin D and liver inflammation. Bar chart for 25-hydroxy vitamin D and portal inflammation (A), lobular inflammation (B), interface hepatitis (C) and scatter plots for 25-hydroxy vitamin D and normalized ALT (D) and AST (E). Mean concentration and 95% confidence interval is shown. Statistical significance determined by Student T test, One Way ANOVA and Pearson’s test.

25(OH)D status was also analyzed in relation to the various forms of liver inflammation, i.e. portal inflammation, lobular inflammation and interface hepatitis with no significant differences noted. Also, no correlations were observed between 25(OH)D concentrations and serum concentrations of normalized alanine aminotransferase (nALT) and normalized aspartate aminotransferase (nAST), i.e. the ratio between measured ALT or AST and the upper limit of normal, Fig 2A–2E.

### Liver steatosis

**Fig 3:**
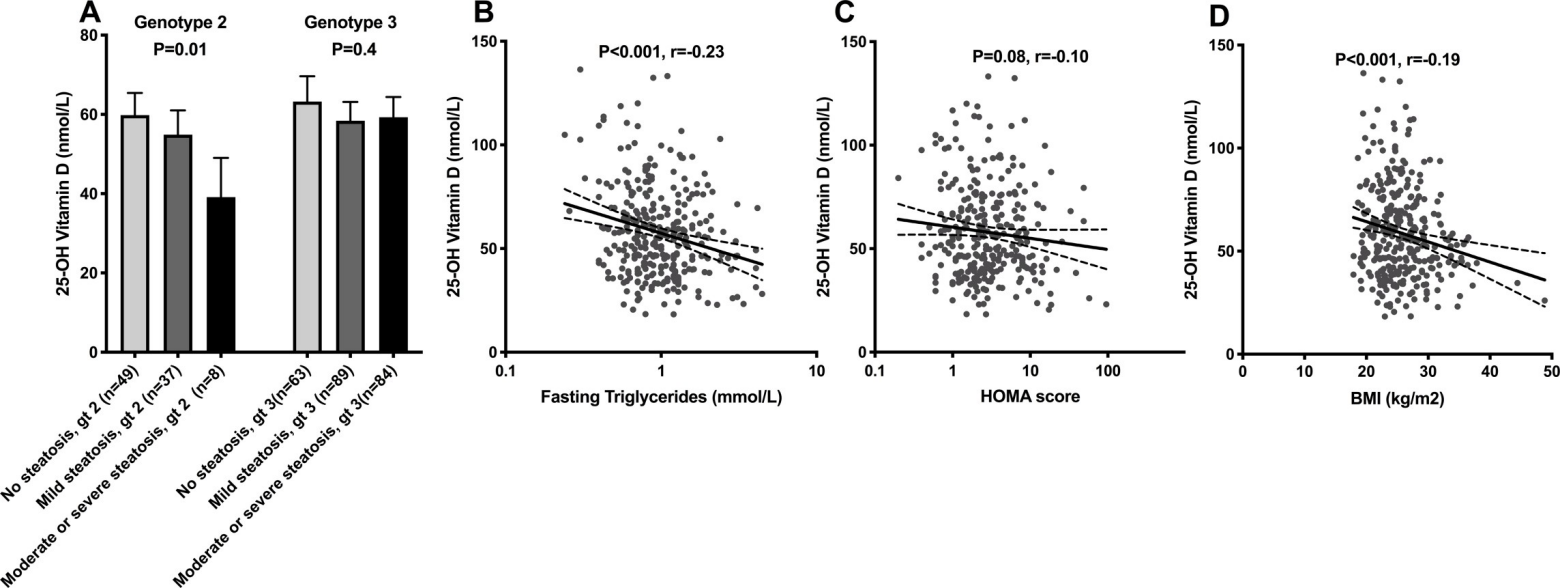
25-hydroxy vitamin D and liver steatosis. Bar chart for 25-hydroxy vitamin D and liver steatosis severity is shown for the different hepatitis C virus genotypes (A). Also scatter plots for 25-hydroxy vitamin D and fasting triglycerides (B), HOMA-score (C) and BMI (D) are shown. Mean concentration and 95% confidence interval is shown. Statistical significance determined by One Way ANOVA and Pearson’s test.

25(OH)D did not differ between the steatosis grade groups (no steatosis, mild steatosis and moderate-to-severe steatosis) when analyzing all the patients. Since infection with HCV genotype 3 is associated with markedly more pronounced steatosis, HCV genotypes 2 and 3 where analyzed separately. In a one-way analysis of variance (ANOVA) for patient infected with HCV genotype 2, there was a significant difference in 25(OH)D concentration between steatosis grade groups (F (2,91) = 4.4, p = 0.01). The difference was only significant when comparing patients with no steatosis and moderate-to-severe steatosis, (60 ±20 vs. 39 ±12 nmol/L, p = 0.01), but a similar, non-significant trend was noted between mild steatosis and moderate-to-severe steatosis, (55 ±18 vs. 39 ±12 nmol/L, p = 0.08). No such difference was observed among genotype 3 infected patients, Fig 3A. Vitamin D levels demonstrated clear correlations with BMI and fasting triglycerides, whereas only a non-significant trend was observed for homeostatic model assessment (HOMA) score, Fig 3B–3D.

### Seasonality

**Fig 4:**
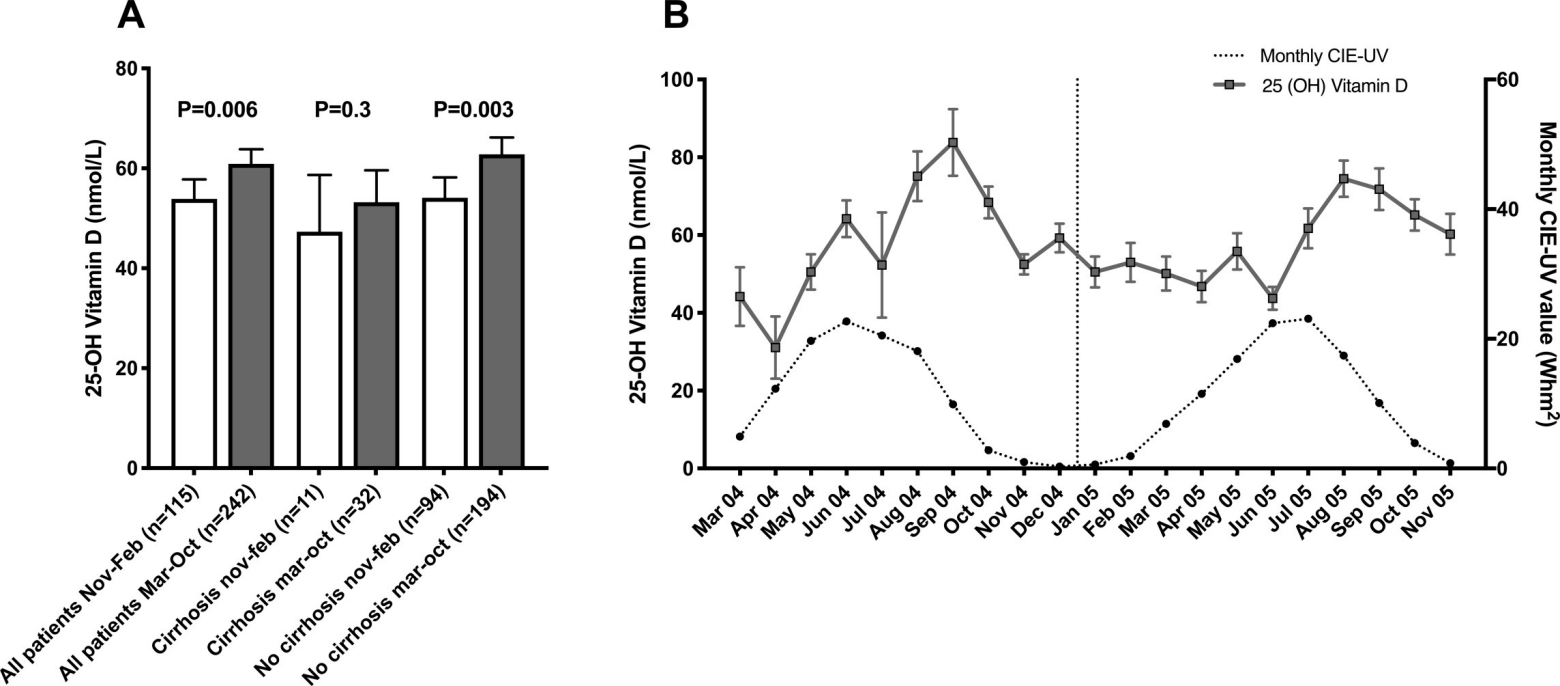
25-hydroxy vitamin D and season. Bar chart for 25-hydroxy vitamin D and seasonal variation shown for all patients, cirrhotics and non cirrhotics (A) and monthly variations together with UVB radiation are shown (B). Mean concentration and 95% confidence interval is shown for (A) and mean and standard error of mean for (B). Statistical significance determined by Student T test.

Based on data from the Swedish metrological and hydrological institute, a high UV exposure period from March to October and a low sun exposure period from November to February were chosen. 25(OH)D levels were significantly higher in patients sampled March-October compared to November-February (61 ±23 vs. 54 ±21 nmol/L, p<0.01), and this was also significant for non-cirrhotic patients (63 ±24 vs. 54 ±20 nmol/L, p<0.01). Also, a similarly non-significant difference was observed for the cirrhotic patients (53 ±18 vs. 47 ±17 nmol/L, p = 0.35), Fig 4A. When plotting 25 (OH) values and UVB radiation at time of sampling (monthly UV radiation values in Norrköping, Sweden), as expected, vitamin D values increased during the summer months and peaked in late summer and early fall, Fig 4B. A majority of patients were sampled during the period with high UV radiation.

### Virology

No difference in serum 25(OH)D concentrations were observed between patients infected with HCV genotype 2 as compared to genotype 3 (56 ±19 vs. 60 ±24 nmol/L, p = 0.18), and no correlation with baseline HCV RNA level was observed (r = 0.66, p = 0.23). Likewise, there were no significant differences in the likelihood of achieving a cure, i.e. sustained virologic response (SVR), following interferon-based HCV therapy in the sufficient, potential risk of insufficiency and higher risk of insufficiency groups, Table 1. Patients achieving SVR also had similar serum 25(OH)D concentration compared to treatment failures (60 ±23 vs 58 ±22 nmol/L, p = 0.52).

### Multiple regression analysis for 25(OH)D concentration and fibrosis

Linear and multiple linear regression was calculated to further analyze associations with 25(OH)D concentration and fibrosis. Variables with a *p-value* of less than 0.1 in the linear regression were included in the multiple regression analysis. For 25(OH)D concentration included variables were BMI, ethnicity, fibrosis and season at sampling. The highest significant standardized beta coefficient in the multiple regression (*β-value)*, indicating the highest impact on variations in 25(OH)D concentration was seen for BMI, but this value was similar to those for ethnicity and season at sampling, whereas the significant association between fibrosis and 25(OH)D concentration vanished in this analysis. 25(OH)D concentration was likewise not significantly associated with fibrosis in the multiple regression analysis for explaining fibrosis severity, Table 2.

## Discussion and conclusion

The main findings in this study were: i) Liver fibrosis correlated weakly with 25(OH)D concentration and cirrhotic patients had significantly lower levels of 25(OH)D in univariate analyses, although this significance was lost in multivariate analyses. ii) Forty percent of Scandinavian patients with chronic HCV genotype 2 or 3 infection are at potential risk of vitamin D deficiency (<50 nmol/L of 25(OH)D) and 78% had values <75nmol/L but high risk of deficiency (<30 nmol/L) was uncommon (≈6%). iii) 25(OH)D levels were highly significantly and independently inversely associated with BMI and non-Caucasian ethnicity. iv) There are significant seasonal variations in 25(OH) D concentrations in HCV genotype 2 or 3 infected patients in northern Europe.

In the present study, hepatic fibrosis stage was assessed by means of liver biopsies centrally evaluated using the Ishak protocol by two experienced physicians reaching consensus scores, which likely yielded high consistency, as previously documented [26]. Despite this and the statistical power of 331 enrolled patients, fibrosis stage did not independently correlate with 25(OH)D concentrations. Cirrhotic patients had significantly lower vitamin D levels but when accounting for covariates, e.g. BMI, in multivariate analyses, the significant association between cirrhosis and 25(OH)D levels observed in univariate analyses vanished. However, it should be noted that despite the findings in the present study, there may be an association in patients with decompensated cirrhosis (Child-Pugh B or C) as previously reported [27], or in non-Caucasian patients, who have an established higher risk of vitamin D deficiency. The former group was excluded from enrollment in the NORDynamIC study as interferon therapy is contraindicated in decompensated cirrhosis [28], and the latter group was relatively small (16 of 331 patients were non-Caucasian in the study) making it challenging to obtain robust statistical results. In less severe liver disease, the hydroxylation to 25(OH)D in the liver might still be sufficient to maintain serum concentrations of 25(OH)D, and an effect would likely be more evident when liver function is failing as in patients with decompensated cirrhosis. Thus, our results might have differed if patients with Child-Pugh B or C cirrhosis also had been enrolled in the study, but aside from being interferon-intolerant, decompensated cirrhotic patients often have contraindications to ordinary percutaneous liver biopsy, e.g. severe thrombocytopenia or coagulopathy [29].

A recent study evaluating the 25(OH)D levels in healthy Swedish blood donors reported a mean concentration of 61 nmol/L with high seasonal variations, and also noted that 35% had values <50 nmol/L and 75% <75 nmol/L [30]. In the present study, similar levels were observed indicating that patients enrolled in the NORDynamIC study, despite approximately half having significant liver fibrosis (Ishak stage ≥3), did not differ substantially from healthy blood donors regarding vitamin D status. However, it should be noted that in northern Europe UVB radiation differs over the year, with exceptionally low values observed from November until February. During this season, humans rely on stored reserves, dietary intake, and/or supplementation of vitamin D. The result of the present study is somewhat alarming as 5% of patients had levels <25 nmol/L during this period, and may have benefitted from supplementation, irrespective of symptoms, according to current Swedish guidelines [31]. This of course also applies to healthy Swedish blood donors where a study showed that levels <25 nmol/L were around 9% during this same period [30]. In contrast to these findings, another study from northern Sweden reported that only 0.7% had values <25 nmol/L, albeit that all participants were sampled from January to April 2009 [32]. The latter study used high pressure liquid chromatography with tandem mass spectrometry, which is considered the golden standard. Chemiluminiscence immunoassays, as used in this study and in many routine laboratories, tend to overestimate the proportion of 25(OH)D deficient patients, which is important to consider when interpreting the high percentage of low vitamin D levels [33]. The comparable proportion of 25(OH)D deficiency in our study patients compared to blood donors do not support general screening for deficiency among HCV infected patients with compensated liver disease.

Lower vitamin D levels have been seen in patients with autoimmune hepatitis with marked inflammation in liver biopsies [19]. Vitamin D also appears to have anti-inflammatory properties [34]. No such correlation between 25(OH)D and inflammation grade or any significant differences in 25(OH)D depending on grade of interface hepatitis, lobular inflammation, portal inflammation or elevated liver enzymes were detectable in the present study. The proposed anti-inflammatory effect of vitamin D thus does not seem to have any major impact on inflammation driven by chronic HCV genotype 2 or 3 infection.

Steatosis was analyzed based on HCV genotype, as HCV genotype 3 infection is known to be associated with more severe steatosis [35, 36]. HCV genotype 2 infected patients with moderate-to-severe steatosis had significantly lower vitamin D levels compared to patients with no steatosis. However, only eight HCV genotype 2 infected patients had moderate-to-severe steatosis, and thus this result should be interpreted cautiously. This difference was not observed in genotype 3 infected patients likely due to the strong correlation between HCV viral load and steatosis in genotype 3. Lower levels of 25(OH)D have been associated with non-alcoholic steatohepatitis (NASH) as well as with non-alcoholic fatty liver disease (NAFLD), also when accounting for features of the metabolic syndrome [10].

Polymorphisms in rs2228570 (Fok1), which causes a threonine-methionine change in the VDR, and rs7975232 (ApaI), located in the 3’ untranslated region of the VDR gene, were not associated with 25(OH)D concentration or fibrosis. As polymorphisms in both these SNPs predominantly affect the function and abundance of the receptor for vitamin D, the lack of association with serum 25(OH)D concentrations in the present study was not surprising and corroborate prior observations [37], although contrasting with reported observations in a pediatric cohort [38]. rs7975232 (ApaI) CC allele configuration alone and in combination with rs1544410 (BsmI) and rs7311236 (TaqI) in the CCA haplotype, has also been shown to be associated with fibrosis progression in patient with chronic HCV [20]. rs10877012 is situated in the CYP27B-1260 gene promotor and responsible for conversion of 25(OH)D to active 1,25(OH)D. These SNPs have been linked to 1,25(OH)D concentration [23]. No significant associations were noted between these SNPs and 25(OH)D or fibrosis, in contrast to a previous report [23]. These three SNPs were chosen because they are thought to have an effect downstream of 25(OH)D through either influencing the receptor or the activation of vitamin D, but they, like 25(OH)D concentration, all failed to have any independent association with fibrosis stage. There are several SNPs involved in vitamin D signaling and metabolism, and a more thorough screening would have been beneficial. For example, we only analyzed one of the SNPs in the ApaI, BsmI and TaqI CCA haplotype, namely ApaI. However, recently BsmI and TaqI, but not ApaI was shown to associate with fibrosis indices in patients with chronic HCV [39].

Vitamin D levels were highly significantly and independently inversely correlated to BMI, and the impact of obesity on 25(OH)D concentration overshadowed other baseline characteristics, including liver fibrosis. The importance of obesity for vitamin D deficiency has been previously well-established [40] and it has been suggested that lower vitamin D concentrations in obese patients may be attributable to lower dietary intake [41], decreased sun exposure [42], sequestering in adipose tissue [43, 44], and/or volumetric dilution [45]. The association between obesity and cirrhosis in the absence of other causes of liver disease has also been demonstrated [46, 47], although the underlying mechanisms remains to be fully elucidated. Other important determinants of 25(OH)D concentration were ethnicity and season at sampling, in accordance with prior reports [48, 49].

There are some limitations in this study. Most important is that patients with decompensated liver cirrhosis were not included because of interferon intolerance, as a potential effect of liver disease on 25(OH)D concentration most likely would be observed in such patients. The study also is cross-sectional, and a longitudinal study would offer more reliable data especially in a region like Scandinavia were UV-radiation differ much throughout the year. The prevalence of vitamin D deficiency was relatively low in our study, and if deficiency drives an acceleration in fibrosis progression, this could explain the lack of association between fibrosis and 25(OH)D, although this is controversial [50]. In order to address the potential relation between vitamin D and liver fibrosis, large longitudinal prospective randomized trials spanning over decades with and without intervention with vitamin D supplementation would be required.

Thus, in conclusion the present study demonstrated, as expected, significant seasonal variations in 25-hydroxy vitamin D concentrations in northern Europe, and that higher BMI and non-Caucasian ethnicity were independently and strongly associated with lower serum vitamin D concentrations, suggesting that they may have a stronger negative impact on 25(OH)D levels than liver histopathology and other baseline characteristics in HCV genotype 2 or 3 infected patients with compensated liver disease.

## Supporting information

S1 Dataset Data used in this publication. (XLSX)

S1 Table Associations of baseline characteristics with 25 (OH) D concentrations and fibrosis. Analysis only including Caucasian patients.
(DOCX)

## References

[1] Holick MF. Resurrection of vitamin D deficiency and rickets. J Clin Invest. 2006;116(8):2062–72. 10.1172/JCI29449 16886050 PMC1523417

[2] Rafiq S, Jeppesen PB. Body Mass Index, Vitamin D, and Type 2 Diabetes: A Systematic Review and Meta-Analysis. Nutrients. 2018;10(9). 10.3390/nu10091182 . 30154381 PMC6164132

[3] Barnard K, Colon-Emeric C. Extraskeletal effects of vitamin D in older adults: cardiovascular disease, mortality, mood, and cognition. Am J Geriatr Pharmacother. 2010;8(1):4–33. 10.1016/j.amjopharm.2010.02.004 . 20226390

[4] Rosen CJ, Adams JS, Bikle DD, Black DM, Demay MB, Manson JE, et al The nonskeletal effects of vitamin D: an Endocrine Society scientific statement. Endocr Rev. 2012;33(3):456–92. 10.1210/er.2012-1000 22596255 PMC3365859

[5] Webb AR, Kline L, Holick MF. Influence of season and latitude on the cutaneous synthesis of vitamin D3: exposure to winter sunlight in Boston and Edmonton will not promote vitamin D3 synthesis in human skin. J Clin Endocrinol Metab. 1988;67(2):373–8. 10.1210/jcem-67-2-373 . 2839537

[6] Bikle DD. Vitamin D metabolism, mechanism of action, and clinical applications. Chem Biol. 2014;21(3):319–29. 10.1016/j.chembiol.2013.12.016 24529992 PMC3968073

[7] Holick MF. Vitamin D status: measurement, interpretation, and clinical application. Ann Epidemiol. 2009;19(2):73–8. 10.1016/j.annepidem.2007.12.001 18329892 PMC2665033

[8] Ross AC, Manson JE, Abrams SA, Aloia JF, Brannon PM, Clinton SK, et al The 2011 report on dietary reference intakes for calcium and vitamin D from the Institute of Medicine: what clinicians need to know. J Clin Endocrinol Metab. 2011;96(1):53–8. 10.1210/jc.2010-2704 21118827 PMC3046611

[9] Holick MF, Binkley NC, Bischoff-Ferrari HA, Gordon CM, Hanley DA, Heaney RP, et al Evaluation, treatment, and prevention of vitamin D deficiency: an Endocrine Society clinical practice guideline. J Clin Endocrinol Metab. 2011;96(7):1911–30. 10.1210/jc.2011-0385 . 21646368

[10] Liangpunsakul S, Chalasani N. Serum vitamin D concentrations and unexplained elevation in ALT among US adults. Dig Dis Sci. 2011;56(7):2124–9. 10.1007/s10620-011-1707-x 21503677 PMC3644216

[11] Prietl B, Treiber G, Pieber TR, Amrein K. Vitamin D and immune function. Nutrients. 2013;5(7):2502–21. 10.3390/nu5072502 23857223 PMC3738984

[12] Gal-Tanamy M, Bachmetov L, Ravid A, Koren R, Erman A, Tur-Kaspa R, et al Vitamin D: an innate antiviral agent suppressing hepatitis C virus in human hepatocytes. Hepatology. 2011;54(5):1570–9. 10.1002/hep.24575 . 21793032

[13] Huang JF, Ko YM, Huang CF, Yeh ML, Dai CY, Hsieh MH, et al 25-Hydroxy vitamin D suppresses hepatitis C virus replication and contributes to rapid virological response of treatment efficacy. Hepatol Res. 2017;47(13):1383–9. 10.1111/hepr.12878 . 28225575

[14] Villar LM, Del Campo JA, Ranchal I, Lampe E, Romero-Gomez M. Association between vitamin D and hepatitis C virus infection: a meta-analysis. World J Gastroenterol. 2013;19(35):5917–24. 10.3748/wjg.v19.i35.5917 24124339 PMC3793147

[15] Loftfield E, O'Brien TR, Pfeiffer RM, Howell CD, Horst R, Prokunina-Olsson L, et al Vitamin D Status and Virologic Response to HCV Therapy in the HALT-C and VIRAHEP-C Trials. PLoS One. 2016;11(11):e0166036 10.1371/journal.pone.0166036 27832143 PMC5104464

[16] Hoan NX, Tong HV, Song LH, Meyer CG, Velavan TP. Vitamin D deficiency and hepatitis viruses-associated liver diseases: A literature review. World J Gastroenterol. 2018;24(4):445–60. 10.3748/wjg.v24.i4.445 29398866 PMC5787780

[17] Dadabhai AS, Saberi B, Lobner K, Shinohara RT, Mullin GE. Influence of vitamin D on liver fibrosis in chronic hepatitis C: A systematic review and meta-analysis of the pooled clinical trials data. World J Hepatol. 2017;9(5):278–87. 10.4254/wjh.v9.i5.278 28261385 PMC5316848

[18] Konstantakis C, Tselekouni P, Kalafateli M, Triantos C. Vitamin D deficiency in patients with liver cirrhosis. Ann Gastroenterol. 2016;29(3):297–306. 10.20524/aog.2016.0037 27366029 PMC4923814

[19] Efe C, Kav T, Aydin C, Cengiz M, Imga NN, Purnak T, et al Low serum vitamin D levels are associated with severe histological features and poor response to therapy in patients with autoimmune hepatitis. Dig Dis Sci. 2014;59(12):3035–42. 10.1007/s10620-014-3267-3 . 25002309

[20] Baur K, Mertens JC, Schmitt J, Iwata R, Stieger B, Eloranta JJ, et al Combined effect of 25-OH vitamin D plasma levels and genetic vitamin D receptor (NR 1I1) variants on fibrosis progression rate in HCV patients. Liver Int. 2012;32(4):635–43. 10.1111/j.1478-3231.2011.02674.x . 22151003

[21] Baur K, Mertens JC, Schmitt J, Iwata R, Stieger B, Frei P, et al The vitamin D receptor gene bAt (CCA) haplotype impairs the response to pegylated-interferon/ribavirin-based therapy in chronic hepatitis C patients. Antivir Ther. 2012;17(3):541–7. 10.3851/IMP2018 . 22300961

[22] Uitterlinden AG, Fang Y, Van Meurs JB, Pols HA, Van Leeuwen JP. Genetics and biology of vitamin D receptor polymorphisms. Gene. 2004;338(2):143–56. 10.1016/j.gene.2004.05.014 . 15315818

[23] Lange CM, Bojunga J, Ramos-Lopez E, von Wagner M, Hassler A, Vermehren J, et al Vitamin D deficiency and a CYP27B1-1260 promoter polymorphism are associated with chronic hepatitis C and poor response to interferon-alfa based therapy. J Hepatol. 2011;54(5):887–93. 10.1016/j.jhep.2010.08.036 . 21145801

[24] Lagging M, Langeland N, Pedersen C, Farkkila M, Buhl MR, Morch K, et al Randomized comparison of 12 or 24 weeks of peginterferon alpha-2a and ribavirin in chronic hepatitis C virus genotype 2/3 infection. Hepatology. 2008;47(6):1837–45. 10.1002/hep.22253 . 18454508

[25] W. J. UV-radiation 1983–2003 measured at Norrköping, Sweden. Theoretical and Applied Climatology. 2006;V.83(No.1-4):59–76. 10.1007/s00704-005-0160-1

[26] Westin J, Lagging LM, Wejstal R, Norkrans G, Dhillon AP. Interobserver study of liver histopathology using the Ishak score in patients with chronic hepatitis C virus infection. Liver. 1999;19(3):183–7. Epub 1999/07/08. 10.1111/j.1478-3231.1999.tb00033.x . 10395036

[27] Malham M, Jorgensen SP, Ott P, Agnholt J, Vilstrup H, Borre M, et al Vitamin D deficiency in cirrhosis relates to liver dysfunction rather than aetiology. World J Gastroenterol. 2011;17(7):922–5. 10.3748/wjg.v17.i7.922 21412501 PMC3051142

[28] Fisher L, Fisher A. Vitamin D and parathyroid hormone in outpatients with noncholestatic chronic liver disease. Clin Gastroenterol Hepatol. 2007;5(4):513–20. 10.1016/j.cgh.2006.10.015 . 17222588

[29] Bircher J, Zimmermann A. [Indications and contraindications of liver biopsy]. Schweiz Med Wochenschr. 1978;108(12):462–5. Epub 1978/03/25. . 635506

[30] Klingberg E, Olerod G, Konar J, Petzold M, Hammarsten O. Seasonal variations in serum 25-hydroxy vitamin D levels in a Swedish cohort. Endocrine. 2015;49(3):800–8. 10.1007/s12020-015-0548-3 25681052 PMC4512566

[31] Lorentzon M. D-vitaminbehandling och skeletthälsa–svenska riktlinjer behövs. Rekommendationer från Svenska osteoporossällskapets kliniska expertgrupp. Läkartidningen. 2014;2014;111:CW6C. 25325102

[32] Ramnemark A, Norberg M, Pettersson-Kymmer U, Eliasson M. Adequate vitamin D levels in a Swedish population living above latitude 63 degrees N: The 2009 Northern Sweden MONICA study. Int J Circumpolar Health. 2015;74:27963 10.3402/ijch.v74.27963 28417824 PMC4432023

[33] al He. New vitamin D blood tests are often highly inaccurate, researchers say. Media release summarizing presentation by Holmes et al at the June 2012 Endocrine Society’s 94th Annual Meeting in Houston, TX. Available at: http://www.eurekalert.org/pub_releases/2012-06/tes-tnv062412.php. [cited 2019 May 20].

[34] Kitson MT, Roberts SK. D-livering the message: the importance of vitamin D status in chronic liver disease. J Hepatol. 2012;57(4):897–909. 10.1016/j.jhep.2012.04.033 . 22634121

[35] Westin J, Lagging M, Dhillon AP, Norkrans G, Romero AI, Pawlotsky JM, et al Impact of hepatic steatosis on viral kinetics and treatment outcome during antiviral treatment of chronic HCV infection. J Viral Hepat. 2007;14(1):29–35. 10.1111/j.1365-2893.2006.00777.x . 17212641

[36] Westin J, Nordlinder H, Lagging M, Norkrans G, Wejstal R. Steatosis accelerates fibrosis development over time in hepatitis C virus genotype 3 infected patients. J Hepatol. 2002;37(6):837–42. Epub 2002/11/26. 10.1016/s0168-8278(02)00299-4 . 12445426

[37] Karonova T, Grineva E, Belyaeva O, Bystrova A, Jude EB, Andreeva A, et al Relationship Between Vitamin D Status and Vitamin D Receptor Gene Polymorphisms With Markers of Metabolic Syndrome Among Adults. Front Endocrinol (Lausanne). 2018;9:448 Epub 2018/09/01. 10.3389/fendo.2018.00448 30166978 PMC6106967

[38] Karpinski M, Galicka A, Milewski R, Popko J, Badmaev V, Stohs SJ. Association between Vitamin D Receptor Polymorphism and Serum Vitamin D Levels in Children with Low-Energy Fractures. J Am Coll Nutr. 2017;36(1):64–71. Epub 2017/01/10. 10.1080/07315724.2016.1218803 . 28067591

[39] Scalioni LP, Santos BRD, Spritzer PM, Villela-Nogueira CA, Laura Lewis-Ximenez L, Pollo-Flores P, et al Impact of vitamin D receptor and binding protein gene polymorphisms in clinical and laboratory data of HCV patients: Cross sectional study. Medicine (Baltimore). 2018;97(8):e9881 10.1097/MD.0000000000009881 29465575 PMC5842007

[40] Vanlint S. Vitamin D and obesity. Nutrients. 2013;5(3):949–56. Epub 2013/03/23. 10.3390/nu5030949 23519290 PMC3705328

[41] Kamycheva E, Joakimsen RM, Jorde R. Intakes of calcium and vitamin d predict body mass index in the population of Northern Norway. J Nutr. 2003;133(1):102–6. Epub 2003/01/07. 10.1093/jn/133.1.102 . 12514276

[42] Kull M, Kallikorm R, Lember M. Body mass index determines sunbathing habits: implications on vitamin D levels. Intern Med J. 2009;39(4):256–8. Epub 2009/05/01. 10.1111/j.1445-5994.2009.01900.x . 19402866

[43] Rosenstreich SJ, Rich C, Volwiler W. Deposition in and release of vitamin D3 from body fat: evidence for a storage site in the rat. J Clin Invest. 1971;50(3):679–87. Epub 1971/03/01. 10.1172/JCI106538 4322721 PMC291976

[44] Blum M, Dolnikowski G, Seyoum E, Harris SS, Booth SL, Peterson J, et al Vitamin D(3) in fat tissue. Endocrine. 2008;33(1):90–4. Epub 2008/03/14. 10.1007/s12020-008-9051-4 18338271 PMC2839878

[45] Drincic AT, Armas LA, Van Diest EE, Heaney RP. Volumetric dilution, rather than sequestration best explains the low vitamin D status of obesity. Obesity (Silver Spring). 2012;20(7):1444–8. Epub 2012/01/21. 10.1038/oby.2011.404 . 22262154

[46] Clain DJ, Lefkowitch JH. Fatty liver disease in morbid obesity. Gastroenterol Clin North Am. 1987;16(2):239–52. Epub 1987/06/01. . 3319904

[47] Angulo P, Keach JC, Batts KP, Lindor KD. Independent predictors of liver fibrosis in patients with nonalcoholic steatohepatitis. Hepatology. 1999;30(6):1356–62. 10.1002/hep.510300604 . 10573511

[48] Lundstrom P, Caidahl K, Eriksson MJ, Fritz T, Krook A, Zierath JR, et al Changes in Vitamin D Status in Overweight Middle-Aged Adults with or without Impaired Glucose Metabolism in Two Consecutive Nordic Summers. J Nutr Metab. 2019;2019:1840374 10.1155/2019/1840374 30944737 PMC6421780

[49] Barebring L, Schoenmakers I, Glantz A, Hulthen L, Jagner A, Ellis J, et al Vitamin D Status during Pregnancy in a Multi-Ethnic Population-Representative Swedish Cohort. Nutrients. 2016;8(10). 10.3390/nu8100655 27782070 PMC5084041

[50] Keane JT, Elangovan H, Stokes RA, Gunton JE. Vitamin D and the Liver-Correlation or Cause? Nutrients. 2018;10(4). 10.3390/nu10040496 29659559 PMC5946281

