# Supplementary material for: The relation of 25-hydroxy vitamin D concentrations to liver histopathology, seasonality and baseline characteristics in chronic hepatitis C virus genotype 2 or 3 infection
Source: PLoS One. 2020 Aug 21;15(8):e0237840. doi: 10.1371/journal.pone.0237840 (PMC7442235; doi:10.1371/journal.pone.0237840)
Supplement: S1 Table — Analysis only including Caucasian patients. (DOCX) [file pone.0237840.s002.docx]

**S1 Table. Associations of Baseline Characteristics with 25 (OH) D concentrations and fibrosis. Analysis only including Caucasian patients.**

| Feature | **Associations of Baseline Characteristics and 25 (OH) D concentrations** | | | | | | **Associations of Baseline Characteristics and fibrosis** | | | | | |
| --- | --- | --- | --- | --- | --- | --- | --- | --- | --- | --- | --- | --- |
|  | B-value | β-value | *P-value* | Adjusted B-value | Adjusted β-value | Adjusted *P-value* | B-value | β-value | *P-value* | *Adjusted B-value* | Adjusted β-value | Adjusted *P-value* |
| Epidemiological features |  |  |  |  |  |  |  |  |  |  |  |  |
| Age, years | -0.07 | -0.03 | 0.57 |  |  |  | 0.06 | 0.44 | **<0.001** | 0.05 | 0.33 | **<0.001** |
| Sex (female) | 0.01 | 0.00 | 1.0 |  |  |  | -0.38 | -0.13 | **0.03** | -0.11 | -0.04 | 0.5 |
| BMI, kg/m2 | -1.07 | -0.21 | **0.0002** | -1.04 | -0.21 | **0.0003** | 0.06 | 0.19 | **0.001** | 0.05 | 0.15 | **0.005** |
| Characteristics of HCV infection |  |  |  |  |  |  |  |  |  |  |  |  |
| HCV-RNA at baseline, log10 IU/mL | 2.16 | 0.08 | 0.14 |  |  |  | 0.34 | 0.20 | **<0.001** | 0.08 | 0.04 | 0.4 |
| HCV genotype, genotype 2/3 | 4.98 | 0.10 | **0.07** | 5.97 | 0.12 | **0.03** | -0.10 | -0.03 | 0.6 |  |  |  |
| Liver firosis Ishak, score | -1.67 | -0.11 | **≤0.05** | -1.37 | -0.09 | 0.11 |  |  |  |  |  |  |
| Liver Ishak inflammation, sum of score | -1.01 | -0.09 | 0.13 |  |  |  | 0.55 | 0.71 | **<0.001^a^** |  |  |  |
| Liver Ishak steatosis, score | -1.80 | -0.08 | 0.16 |  |  |  | 0.35 | 0.24 | **<0.001** | 0.03 | 0.02 | 0.7 |
| APRI-score (log10) | -2.10 | -0.03 | 0.57 |  |  |  | 2.06 | 0.50 | **<0.001** | 1.68 | 0.41 | **<0.001** |
| Season |  |  |  |  |  |  |  |  |  |  |  |  |
| Sunny season at sampling | 8.58 | 0.18 | **0.001** | 9.35 | 0.2 | **0.001** |  |  |  |  |  |  |
| Serum 25 (OH)D levels |  |  |  |  |  |  |  |  |  |  |  |  |
| Serum 25 (OH)D, nmol/L |  |  |  |  |  |  | **-0.01** | **-0.11** | **≤0.05** | -0.00 | -0.03 | 0.6 |
| HCV, hepatitis C virus; APRI, sat to platelet ratio. ^a^Excluded from multiple regression due to multicollinearity. |  |  |  |  |  |  |  |  |  |  |  |  |
